# Population-Based Assessment of Phenoconversion Potential in Switzerland: A Claims Data Study of Key Drug-Metabolizing Enzymes and Transporters

**DOI:** 10.2147/PGPM.S586384

**Published:** 2026-05-20

**Authors:** Marietta M Roth, Aline Bencastro, Christoph R Meier, Carola A Huber, Henriette E Meyer zu Schwabedissen, Samuel S Allemann, Cornelia Schneider

**Affiliations:** 1 Basel Pharmacoepidemiology Unit, Division of Clinical Pharmacy and Epidemiology, Department of Pharmaceutical Sciences, University of Basel, Basel, Switzerland; 2 Hospital Pharmacy, University Hospital Basel, Basel, Switzerland; 3 Department of Health Sciences, Helsana Group, Zürich, Switzerland; 4 Biopharmacy, Department of Pharmaceutical Sciences, University of Basel, Basel, Switzerland; 5 Pharmaceutical Care, Department of Pharmaceutical Sciences, University of Basel, Basel, Switzerland

**Keywords:** drug-drug interaction, drug-drug-gene interaction, phenoconversion

## Abstract

**Purpose:**

Drug-drug-gene interactions can alter drug exposure and thereby increase the risk of clinically relevant outcomes, such as concentration-dependent toxicity (eg, tacrolimus toxicity in the context of altered CYP3A4/5 activity) or reduced treatment effectiveness. Despite their emerging relevance in clinical research, drug-drug-gene interactions remain understudied and are often ignored in clinical practice. Our objective was to assess the risk of phenoconversion by identifying potential drug-drug-gene interactions involving the transporters OATP1B1 and BCRP and the enzymes CYP2B6 and CYP3A4/5 in the Swiss population.

**Patients and Methods:**

Using claims data from the Helsana basic health insurance, we identified all persons of all ages with at least one drug claim between 2017 and 2021 and with Helsana basic health insurance coverage for at least one full year. For the five-year analysis, only persons with insurance for the entire five-year period were included. Within this study population, we assessed and ranked the frequency of potential drug-drug-gene interactions of a pharmacogenetic substrate and an inhibitor/inducer of OATP1B1, BCRP, CYP2B6, or CYP3A4/5. Potential drug-drug-gene interactions were defined as the co-occurrence of a pharmacogenetic substrate and an inhibitor/inducer within a 30- or 5-days window.

**Results:**

During the entire five-year period, 18’523 (2.1%) and 12’645 (1.4%) individuals were exposed to potential drug-drug-gene interactions using the 30-day and 5-day windows, respectively. Potential drug-drug-gene interactions most frequently involved CYP3A4/5 (81.0% and 85.3%), followed by CYP2B6 (10.9% and 8.7%) and OATP1B1 (8.7% and 13.3%). The top three drug classes involved were nervous system drugs (75.1%), cardiovascular drugs (10.6%), and dermatologicals (4.0%). Quetiapine ranked first in the number of involved drug pairs, with quetiapine – metamizole being the predominant drug pair.

**Conclusion:**

In Switzerland, two out of 100 persons taking drugs metabolized or transported by OATP1B1, BCRP, CYP2B6, and CYP3A4/5 are at potential risk of phenoconversion, predominantly involving CYP3A4/5. These findings quantify real-world phenoconversion potential at the population level and underscore the need for outcome- and genotype-linked studies to determine clinical relevance. As this study was not designed to assess clinical outcomes, future genotype- and outcome-linked investigations are required to determine the actual impact on adverse drug reactions or treatment effectiveness.

## Introduction

The effectiveness of drug response is influenced by various factors, including genetic variability.^1–3^ Genetic variation can alter drug metabolism and response, and preemptive pharmacogenetic (PGx) testing has been shown to reduce the incidence of adverse drug reactions (ADRs).^4–6^ In contrast to the consideration of drug-drug interactions (DDIs) in clinical practice, drug-gene interactions (DGIs) and drug-drug-gene interactions (DDGIs) are rarely taken into account.^7^,^8^ A study using Swiss claims data indicated that up to 75% of individuals are exposed to at least one PGx drug within five years.^7^,^9^ Furthermore, approximately 25% of new prescriptions involve actionable DGIs, and in 5% of cases a change in therapy is required.^9^,^10^ The evaluation of DGIs in clinical practice considers a patients genotype to predict the patient’s phenotype. However, genotype-predicted phenotypes do not always reflect actual metabolic activity. This phenomenon, known as phenoconversion, occurs when non-genetic factors modify the genotype-predicted phenotype, resulting in a discrepancy between the expected and actual drug metabolism.^9^ These factors include comorbidities (eg, cancer), physiological states (eg age, pregnancy), lifestyle (eg, smoking, BMI), and concomitant drug use When caused by co-administered drugs, such cases are referred to as DDGIs.^9–12^

Phenoconversion can alter metabolic capacity in either direction. For instance, inhibitors of cytochrome P450 enzymes can shift metabolism towards a slower phenotype, while inducers may accelerate metabolism. This may lead to ADRs or treatment failure considering that drug metabolism may be involved in bioactivation and inactivation of PGx-drugs.^7^ The impact of such interactions depends on both the individual’s genetic background and the potency of the interacting agent. For instance, individuals who are poor metabolizers of CYP2D6 (PMs) will generally not demonstrate further metabolic reduction when exposed to a CYP2D6 inhibitor, as their enzymatic function is already absent or severely impaired^7^,^11^ Phenoconversion can substantially impact genotype–phenotype correlations and compromise the implementation of pharmacogenetic (PGx) testing in clinical care.^12^ In an Australian cohort, Mostafa et al reported a fivefold increase in the prevalence of CYP2C19 and CYP2D6 PMs due to DDGIs.^13^ A Swiss study that compared measured and genotype predicted CYP phenotypes in patients receiving antidepressants found that the proportion of patients who were classified differently by the two approaches ranged from 33 to 65%, depending on the CYP enzyme. This highlights the fact that the relevance of DDGI varies depending on the isoform.^14^ Dowd et al showed that the DDI risk increased from 26.0% to 49.6% and from 38.7% to 64.4% in the elderly when genetic polymorphisms were simulated.^3^ These findings highlight the value of phenotyping and the emerging significance of DDGIs in personalized pharmacotherapy.

In an ideal setting, drug response would be anticipated before treatment initiation by combining genetic information with relevant environmental and pharmacological factors. At the moment therapeutic drug monitoring (TDM) has been proposed as an appropriate strategy for managing DDIs. However, its utility is limited at therapy initiation since drug levels can only be reliably assessed once a steady state is reached.^12^ Preemptive PGx testing with individual interpretation of potential DDGIs therefore represents a more efficacious and proactive approach to prevention of ADRs even during therapy initiation.^15^ Guidelines are essential for the successful integration of PGx into clinical workflows. To date, the Clinical Pharmacogenetics Implementation Consortium (CPIC) and the Dutch Pharmacogenetics Working Group (DPWG) have published guidelines for over 100 drug-gene pairs, but there are still few explicit recommendations for the management of DDGIs in clinical practice.^16–18^ Although this limitation is increasingly acknowledged, the full extent of phenoconversion remains unknown.

Although CYP2B6 and CYP3A4/5 are pharmacologically and pharmacogenetically well characterized, DDGI-focused epidemiologic studies have more frequently examined CYP2C19, CYP2C9, and CYP2D6.^19–21^ In our study we therefore aimed to address DDGIs focusing on OATP1B1, BCRP, CYP2B6, and CYP3A4/5.

We selected these enzymes and transporters as there are genotype-based dosing guidelines available on ClinPGx (formerly PharmGKB).^20^ With regard to the polymorph enzyme CYP2B6, there is evidence from studies in children and young adults, suggesting actionable phenoconversion by comedication.^22–24^ A similar phenomenon has been reported for adults exposed to inducers or inhibitors of this enzyme. This may also be of relevance for CYP3A4/3A5 considering current recommendations for the use of its substrate drug tacrolimus.^25^,^26^ Here, it was observed, that patients receiving tacrolimus with CYP3A4/5 inhibitors experienced an elevated risk of neurological adverse effects, including falls, morbidity, and mortality. Moreover, we included the efflux transporter BCRP (Gene name: *ABCG2*) and the uptake transporter OATP1B1 (Gene name: *SLCO1B*) in our analysis, as they are not only assumed to be implicated in drug response but also in a common adverse drug event namely the statin-induced muscle toxicity. We quantified potential DDGIs in Switzerland, focusing on the abovementioned enzymes and transporters. Although this analysis does not capture clinical outcomes directly, quantifying population-level exposure to potential DDGIs represents an essential step toward understanding their possible clinical impact and guiding future outcome- and genotype-linked research.

## Materials and Methods

### Data Source and Cohort Selection

In Switzerland, basic health insurance is mandatory for all residents.^27^ The Helsana Group is one of the largest health insurers in Switzerland, covering approximately 15% of the Swiss population across all age groups and all 26 cantons.^28^,^29^ The anonymized Helsana database includes demographic data (eg, age, sex, canton of residence) and pharmacy drug claims (eg, drug purchases with dates and Anatomical Therapeutic Chemical Classification System (ATC) codes), but no clinical parameters such as comorbidities, genotypes, lifestyle, or laboratory results.

### Enzymes and Transporters, Substrates, Inhibitors and Inducers

This study focused on the transporters OATP1B1 (*SLCO1B1*) and BCRP (*ABCG2*) and the enzymes CYP2B6 and CYP3A4/5. Clinically validated substrates were selected based on clinical dosing guidelines listed on PharmGKB as of February 7, 2025, including recommendations from CPIC, DPWG, and others (for a complete list, see Appendix Table 1).^16^,^20^,^30^

Of the 206 identified PGx-relevant drugs, 94 were excluded for lacking actionable recommendations, and two were excluded for referencing only drug classes. After filtering for the four selected targets and excluding unavailable drugs (eg, lovastatin), 12 clinically validated substrates remained. Although PharmGKB listed quetiapine as a CYP3A4 substrate only, it was included here under both CYP3A4 and CYP3A5, due to overlapping metabolism and evidence supporting the role of CYP3A5 in quetiapine metabolism.^31–34^

Inhibitors and inducers were defined based on the FDA’s clinical interaction tables,^35^ the “Carte de Cytochromes” from the University of Geneva,^36^ the Flockhart Table,^37^ and DrugBank Online.^38^ Table 1 presents an overview of all included substrates, inhibitor and inducers. Discrepancies among sources were resolved in favor of DrugBank Online; if the classification remained uncertain, the weaker interaction category was chosen to avoid overestimation. Only in vivo verified and clinically relevant substances available in Switzerland were included in this study. General classes (eg glucocorticoids) and compounds without ATC codes (eg curcuma, grapefruit) were excluded. The ATC codes for systemic versus topical administration and drug combinations containing inhibitors/inducers were also considered (Appendix Table  2). CYP3A4 and CYP3A5 inhibitors/inducers were generally congruent and analyzed together. Amlodipine, listed in the Flockhart Table as a CYP3A5 inhibitor only, was classified as inhibiting both enzymes because of overlapping interaction profiles and literature support.^39–41^

**Table 1:** Substrates, Inhibitors and Inducers

Enzyme			Drug
**OATP1B1**	**Substrates**		Atorvastatin, Fluvastatin, Pitavastatin, Pravastatin, Rosuvastatin, Simvastatin
	**Inhibitor**	**No category**	Atazanavir and Ritonavir, Clarithromycin, Cyclosporine, Darolutamide, Eltrombopag, Gemfibrozil, Lopinavir and Ritonavir, Rifampicin, Ritonavir, Sofosbuvir and Velpatasvir, Sofosbuvir and Velpatasvir and Voxilaprevir, Teriflunomide
	**Inducer**	**No category**	–
**BCRP**	**Substrates**		Allopurinol, Rosuvastatin
	**Inhibitor**	**No category**	Cyclosporine, Darolutamide, Eltrombopag, Febuxostat, Sofosbuvir and Velpatasvir and Voxilaprevir, Teriflunomide
	**Inducer**	**No category**	–
**CYP2B6**	**Substrates**		Efavirenz, Sertraline
	**Inhibitor**	**Strong**	Voriconazole
		**Moderate**	Clopidogrel, Efavirenz, Prasugrel, Sertraline
		**Weak**	Thiotepa
	**Inducer**	**Strong**	Carbamazepine, Metamizole, Phenobarbital, Primidone
		**Moderate**	Artemisinin, Cyclophosphamide, Dexamethasone, Efavirenz, Modafinil, Nevirapine, Rifampicin, Ritonavir
		**Weak**	Isavuconazole, Lorlatinib
		**No category**	Phenytoin
**CYP3A4/5**	**Substrates**		Quetiapine, Tacrolimus
	**Inhibitor**	**Strong**	Atazanavir, Cannabidiol, Ceritinib, Cyclosporine, Clarithromycin, Cobicistat, Darunavir, Fusidic acid, Idelalisib, Itraconazole, Ketoconazole, Miconazole, Posaconazole, Ribociclib, Ritonavir, Silibinin, Voriconazole
		**Moderate**	Amiodarone, Amlodipine, Aprepitant, Ciprofloxacin, Clobazam, Crizotinib, Dasatinib, Desogestrel, Diltiazem, Doxycycline, Dronedarone, Erythromycin, Ethinylestradiol, Fluconazole, Fluoxetine, Fluvoxamine, Gestodene, Imatinib, Isavuconazole, Isoniazid, Letermovir, Lopinavir, Nifedipine, Netupitant, Nilotinib, Quetiapine, Sorafenib, Verapamil
		**Weak**	Ivacaftor, Mifepristone, Omeprazole, Rupacarib
	**Inducer**	**Strong**	Apalutamide, Carbamazepine, Enzalutamide, Felbamate, Hyperici herba, Ivosidenib, Lumacaftor and Ivacaftor, Metamizole, Mitotane, Rifampicin
		**Moderate**	Bosentan, Cenobamate, Cyclophosphamide, Dabrafenib, Dexamethasone, Efavirenz, Elvitegravir, Etravirine, Ifosfamide, Lorlatinib, Nevirapine, Oxcarbazepine, Perampanel, Phenobarbital, Phenytoin, Primidone, Vinblastine
		**Weak**	Betamethasone, Brigotinib, Methylprednisolone, Modafinil, Pioglitazone, Prednisolone, Prednisone, Rifabutin, Rufinamide, Vemurafenib, Zanubrutinib

**Abbreviations**: OATP1B1, organic anion transporting polypeptide 1B1; BCRP, breast cancer resistance protein.

### Statistical Analysis

We analyzed Helsana claims data from January 1, 2017, to December 31, 2021. We chose this period to increase the comparability with previously published data for other enzymes in the Swiss population.^21^ This five-year period enabled both an annual and a longitudinal evaluation of potential drug-drug-gene interactions (DDGIs). DDGIs were defined as the concomitant use of a PGx substrate and an inhibitor or inducer of the same enzyme or transporter (OATP1B1, BCRP, CYP2B6, CYP3A4/5) by the same person within two different time windows (≤ 5 and ≤ 30 days). This dual-window approach allowed us to evaluate the impact of different concomitant use definitions on the prevalence of interactions. Drugs acting as both a substrate and an inhibitor or inducer of the same enzyme were not considered to interact with themselves and thus were not classified as potential DDGI. Study participants were stratified by sex, age group (0–17, 18–39, 40–59, 60–79, 80–99, and 100–119 years), and use of PGx substrates, inhibitors, or inducers. Age was defined as of December 31 of the last study year (2021). We calculated the absolute and relative frequencies, mean number of drugs and claims per person, and mean age. We quantified the number of persons with potential DDGIs, stratified them by sex, and calculated their relative prevalence. We analyzed DDGIs by enzyme/transporters, categorized the involved drugs using ATC codes, and identified the most frequent drug pairs in potential DDGIs. The most frequently involved drugs were determined, as they may disproportionately influence the quantification of phenoconversion risk related to the selected enzymes and transporters.

All analyses were conducted using SAS 9.4 (SAS Institute Inc., Cary, NC) and Excel for Microsoft 365 (version 16.95.1).

### Ethical Approval

According to Article 22 of the Swiss Federal Law on Data Protection, anonymous retrospective studies do not require ethical approval.^42^

## Results

### Study Population Characterization

During the 5-year period 894,748 individuals made 71’451’678 drug claims. Every sixth person (17.6%) claimed at least one PGx substrate, while 25.6% claimed a CYP/transporter inhibitor and 28.4% a CYP inducer, respectively. The mean age of the 5-year study population was 44.5 ± 24.0 years. Between 2017 and 2021, the average number of different drugs claimed was 19.7±16.7. The mean age of women was 46.1 ± 24.3 years compared to 42.7 ± 23.5 years in men, the mean number of drugs was 23.3 ± 17.4 in women and 17.8 ± 14.9 in men. Although most individuals claimed at least one drug, only a few of them were PGx substrates, inhibitors, or inducers (Table 2).

**Table 2:** Characteristics of the Study Population

	Total N (%)	Men N (%)	Women N (%)
**Insured Individuals**	894’748 (100)	425’852 (100)	468’896 (100)
**With Drug Claims**	850’844 (95.1)	396’098 (93.0)	454’746 (97.0)
**OATP1B1**			
**PGx-Substrate**	122’473 (13.7)	65’947 (15.5)	56’526 (12.1)
**Inhibitor**	12’240 (1.4)	5’118 (1.2)	7’122 (1.5)
**BCRP**			
**PGx-Substrate**	63’279 (7.1)	37’248 (8.7)	26’031 (5.6)
**Inhibitor**	2’854 (0.3)	1’740 (0.49	1’114 (0.2)
**CYP2B6**			
**PGx-Substrate**	9’477 (1.1)	3’157 (0.7)	6’320 (1.3)
**Inhibitor or Inducer**	189’194 (21.1)	74’368 (17.5)	114’826 (24.5)
**CYP3A4/5**			
**PGx-Substrate**	27’414 (3.1)	11’098 (2.6)	16’316 (3.5)
**Inhibitor or Inducer**	337’983 (37.8)	137’261 (32.2)	200’722 (42.8)

**Note**: Only claims with a valid ATC code were considered drugs and included in the count of drugs used in the section denominated “All drugs”.

**Abbreviations**: N, number of individuals; PGx, pharmacogenetics; OATP1B1, organic anion transporting polypeptide 1B1; BCRP, breast cancer resistance protein; sd, standard deviation; %, percentage of the category “total” is calculated in relation to the total number of insured individuals, %, percentage of the category “men” and “women” are calculated in relation to the total number of men and women respectively.

### Potential Drug-Drug-Gene Interactions

**Figure 1:**
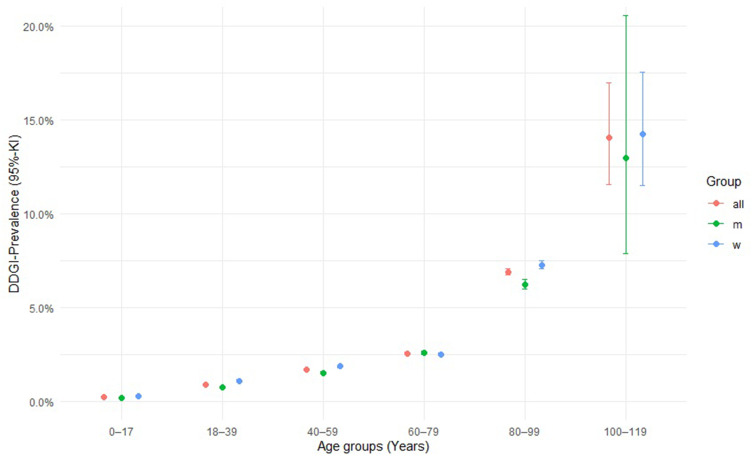
Prevalence of potential DDGIs across age groups and sex.

A total of 18’523 individuals with potential DDGIs, involving a PGx drug and inducer or inhibitor, were registered between 2017 and 2021, using the ±30-days window. A total of 12’645 individuals with potential DDGIs were registered using the ±5-days window. When limiting the interactions to strong inhibitors and inducers and systematically administered drugs, 8’726 individuals with potential DDGIs were registered during the ±30-days window, or 5’652 individuals with potential DDGIs were registered during the ±5-days window. In total, 2.1% (±30-days window) or 1.4% (±5-days window) of individuals were exposed to potential DDGIs. Details on the population with potential DDGIs are shown in Table 3. Age-stratified analyses demonstrated a positive association between age and the prevalence of potential DDGIs as shown in Figure 1. Prevalence increased progressively with age and rose markedly from 60 years onwards, reaching high levels in the oldest age groups. In nearly all age categories, women exhibited higher prevalence estimates than men. If only strong and systemic acting drugs were included, 0.97% (±5-days window) or 0.63% (±30-days window) of individuals were exposed to potential DDGIs. CYP3A4/5 was the most frequently implicated enzyme, accounting for 81.0% (30-day) and 85.3% (5-day) of individuals with potential DDGIs related to any of the four enzymes or transporters. The most frequently involved PGx substrates were quetiapine (CYP3A4/5), sertraline (CYP2B6) and tacrolimus (CYP3A4/5) accounting for 92.2%, 5.9% and 0.9% of potential DDGIs, respectively. The number of individuals with potential DDGIs was higher in women than in men, with 10’984 vs 7’539 during the ±30-day window and 7’496 vs 5’149 during the ±5-day window, respectively. The top 10 drug pairs involved in potential DDGI are listed in Table 4 and Table 5. The most frequently observed potential strong interaction was between quetiapine and metamizole during both time windows, with 754’545 potential DDGI during the ±30-days window, and 154’108 potential DDGI, during the ±5-days window; accounting for more than 50% of all registered instances of potential DDGI. The top three drug classes involved were nervous system drugs (75.1%, quetiapine, metamizole), cardiovascular drugs (10.6%, amlodipine, amiodarone), and dermatologicals (4.0%, imidazole/triazole with corticosteroids, ketoconazole).

**Table 3:** Number of Persons with at Least One Potential DDGI, 2017–2021

2017–2021			30-Day Temporal Window						5-Day Temporal Window					
Enzyme	Category	Subcategory	Total Persons		Men		Women		Total Persons		Men		Women	
			[N]	[%]	[N]	[%]	[N]	[%]	[N]	[%]	[N]	[%]	[N]	[%]
**OATP1B1**	**Total (Substrate-Inhibitor)**	**Total**	1’242	0.14	693	55.80	549	44.20	606	0.07	352	58.09	254	41.91
		**Systemic**	1’184	0.13	675	57.01	509	42.99	578	0.05	341	59.00	237	41.00
**BCRP**	**Total (Substrate – Inhibitor)**	**Total**	539	0.06	376	69.76	163	30.24	374	0.04	271	72.46	103	27.54
		**Systemic**	506	0.06	364	71.94	142	28.06	356	0.04	263	73.88	93	26.12
**CYP2B6**	**Total**	-	2’127	0.24	699	32.86	1’428	67.14	1’120	0.13	355	31.70	765	68.30
	**Substrate – Inhibitor**	**Total**	224	0.03	110	49.11	114	50.89	169	0.02	82	48.52	87	51.48
		**Strong**	1	< 0.01	1	100.00	0	0.00	1	< 0.01	1	100.00	0	0.00
	**Substrate – Inducer**	**Total**	1’969	0.22	618	31.39	1’351	68.61	983	0.11	288	29.30	695	70.70
		**Strong**	1’465	0.16	463	31.60	1’002	68.40	760	0.08	214	28.18	546	71.84
		**Systemic**	1’636	0.18	527	32.21	1’109	67.79	852	0.10	250	29.34	602	70.66
		**Strong systemic**	1’465	0.16	463	31.60	1’002	68.40	760	0.08	214	28.16	546	71.84
**CYP3A4/5**	**Total**	-	15’264	1.70	6’043	39.59	9’221	60.41	10’885	1.22	4’321	39.70	6’564	60.30
	**Substrate – Inhibitor**	**Total**	9’049	1.01	3’649	40.32	5’400	59.68	5’970	0.67	2’432	40.74	3’538	59.26
		**Strong**	3’344	0.37	1’487	44.47	1’857	55.53	1’770	0.20	790	44.63	980	55.37
		**Systemic**	6’915	0.77	2’712	39.21	4’203	60.78	4’671	0.52	1’869	40.01	2’802	59.99
		**Strong systemic**	331	0.04	154	46.53	177	53.47	166	0.02	81	48.80	85	51.20
	**Substrate – Inducer**	**Total**	11’469	1.28	4’515	39.37	6’954	60.63	7’698	0.86	3’020	39.23	4’678	60.77
		**Strong**	6’929	0.77	2’614	37.73	4’315	62.27	4’725	0.53	1’761	37.27	2’964	62.73
		**Systemic**	8’812	0.98	3’484	39.54	5’328	60.46	6’009	0.67	2’362	39.31	3’647	60.69
		**Strong systemic**	6’929	0.77	2’614	37.73	4’315	62.27	4’725	0.53	1’761	37.27	2’964	62.73

**Note**: Specific information on topical, moderate, weak, or no-category inhibitors/inducers are not included in this table, since strong and systemic DDGIs were considered more clinically relevant.

**Abbreviations**: N, number of persons with potential DDGI; %, percentage of persons within the respective subcategory relative to the total number of persons in the study (total persons) or the total number of persons in the subcategory (men, women); OATP1B1, organic anion transporting polypeptide 1B1; BCRP, breast cancer resistance protein; DDGI, drug-drug-gene interaction.

**Table 4:** List of the 10 Most Common Claimed Drug Pairs Involved in Potential DDGIs Within 2017–2021 Using Temporal Window of 30 Days

Ranking	PGx-Substrate	Inhibitor or Inducer	Interaction Type	Enzyme	Total Number of Potential DDGIs [N]	Proportion of Potential DDGIs [%]
**1**	Quetiapine	Metamizole sodium	Strong Induction	CYP3A4/5	754’545	53.70
**2**	Quetiapine	Amlodipine	Moderate Inhibition	CYP3A4/5	250’508	17.83
**3**	Quetiapine	Prednisone	Weak Induction	CYP3A4/5	60’298	4.29
**4**	Sertraline	Metamizole sodium	Strong Induction	CYP2B6	59’230	4.22
**5**	Quetiapine	Amiodarone	Moderate Inhibition	CYP3A4/5	47’263	3.36
**6**	Quetiapine	Imidazoles/triazoles in combination with corticosteroids	Combination	CYP3A4/5	42’203	3.00
**7**	Quetiapine	Prednisolone	Weak Induction	CYP3A4/5	20’728	1.48
**8**	Sertraline	Clopidogrel	Moderate Inhibition	CYP2B6	17’093	1.22
**9**	Quetiapine	Hyperici herba	Strong Induction	CYP3A4/5	13’497	0.96
**10**	Quetiapine	Ketoconazole	Strong Inhibition	CYP3A4/5	13’064	0.93

**Table 5:** List of the 10 Most Common Claimed Drug Pairs Involved in Potential DDGIs Within 2017–2021 Using Temporal Window of 5 days

Ranking	PGx-Substrate	Inhibitor or Inducer	Interaction Type	Enzyme	Total Number of Potential DDGIs [N]	Proportion of Potential DDGIs [%]
**1**	Quetiapine	Metamizole sodium	Strong Induction	CYP3A4/5	154’108	51.64
**2**	Quetiapine	Amlodipine	Moderate Inhibition	CYP3A4/5	52’960	17.75
**3**	Sertraline	Metamizole sodium	Strong Induction	CYP2B6	12’906	4.32
**4**	Quetiapine	Prednisone	Weak Induction	CYP3A4/5	12’293	4.12
**5**	Quetiapine	Amiodarone	Moderate Inhibition	CYP3A4/5	9’697	3.25
**6**	Quetiapine	Imidazoles/triazoles in combination with corticosteroids	Combination	CYP3A4/5	8’793	2.95
**7**	Quetiapine	Prednisolone	Weak Induction	CYP3A4/5	4’423	1.48
**8**	Sertraline	Clopidogrel	Moderate Inhibition	CYP2B6	3’613	1.21
**9**	Quetiapine	Ciprofloxacin	Moderate Inhibition	CYP3A4/5	2’887	0.97
**10**	Quetiapine	Hyperici herba	Strong Induction	CYP3A4/5	2’795	0.94

## Discussion

Between 2017 and 2021, 18’523 individuals (2.1%) were exposed to at least one potential DDGI involving CYP3A4/5, CYP2B6, OATP1B1, or BCRP within a 30-day window. Of those, almost half were exposed to DDGI including strong inducers or inhibitors. For strong interactions, only CYP-associated DDGIs could be calculated, as information on inhibition strength for transporters was missing. Compared with a previous study on the same population, the prevalence was markedly lower. In the previous study 24.8% of individuals were affected by CYP2C19-, CYP2C9-, and CYP2D6-related DDGIs.^21^ This difference likely arises from the lower number of substrates and interacting agents known for the enzymes and transporters analyzed in this study. An Austrian study found that 0.46% of persons were exposed to potential DDGI associated with both OATP1B1 and OATP1B3, and found a slightly higher prevalence than we observed in our population 0.14%.^15^ Despite our low prevalence, these interactions may be clinically relevant. Nonetheless, comparisons with international studies are limited because of the lack of research on DDGIs involving the enzymes and transporters assessed in this study.

For CYP enzymes, DDGIs were more frequently observed than for transporters, with CYP3A4/5 being the most prevalent. There are several explanations for this. These explanations should be interpreted as descriptive and hypothesis-generating, as causal inference is not possible without linked genetic or clinical outcome data. First, we only considered inducers of CYPs, as there are no inducers of the two transporters. In addition, we included a larger number of drugs as CYP3A4/5 inhibitors, which further increased the risk of potential DDGIs. Another plausible contributing factor is the significant involvement of nervous system drugs in CYP-related potential DDGIs, and their complete absence among OATP1B1 and BCRP interacting agents. N-drugs, as anticipated, were highly prevalent among all potential DDGIs, accounting for 62.0% to 82.5% of cases across the 2017–2021 period. This dominance aligns with previous findings on CYP2C9/CYP2C19/CYP2D6-related DDGIs in Switzerland^21^ and international research confirming the central role of N-drugs in PGx-relevant interactions.^3^,^14^,^43^

Beyond this general CYP predominance, distinct sex-specific differences emerged, with women only slightly outnumbering men in the overall population but showing different potential DDGI patterns. CYP2B6- and CYP3A4/5-related DDGIs were more frequent in women, whereas OATP1B1- and BCRP-related interactions predominated in men. Drugs affecting the nervous system are often implicated in CYP-mediated interactions. According to Obsan (2022), in Switzerland 65% of antidepressants and sedatives were prescribed to women compared to 35% prescribed to men.^43^ Other studies have confirmed that women generally receive more prescriptions than men.^44^ This corresponds with the higher CYP2B6- and CYP3A4/5-related DDGIs in our dataset in women compared to men. Women also had a higher mean age (46.1 vs 42.7 years), which increased the risk of polypharmacy and ADRs.^43–46^ These combined factors may explain the higher overall DDGI exposure in women.

The most frequent drug pair in our data was quetiapine and metamizole, which involves the drug metabolizing enzyme CYP3A4/5. Other common agents included sertraline, which was also classified as a PGx substrate in our analysis. Because the number of clinically relevant CYP substrates is limited, commonly used PGx-relevant agents such as quetiapine and sertraline are more likely to appear in potential DDGIs. Quetiapine was the most frequently used agent during the study period. Our analysis grouped CYP3A4 and CYP3A5 together, because of their overlapping profiles, which also applied to quetiapine and amlodipine. While CYP3A4 is the main metabolizer of quetiapine, some studies have suggested a minor role for CYP3A5.^33^,^34^ PharmGKB does not classify quetiapine as a CYP3A5 substrate. Therefore, the quetiapine–amlodipine interaction may have been overrepresented in our results. Excluding these pairs significantly reduced the total number of potential DDGIs. As indicated in a previous study on phenoconversion risk, quetiapine was invariably implicated in the most frequent drug pairs leading to a potential DDGI, which is consistent with our findings. Sertraline was also identified as one of the top ten drug pairs.^21^ Since the drug lists were based on ATC codes, the same active substance might appear multiple times in each table due to drug combinations or different galenic forms. However, merging these results had a minimal impact on overall drug ranking. These findings underscore the phenoconversion risk posed by N-drugs, particularly quetiapine, warranting careful monitoring.

This study used insurance data, which are subject to the limitations of claims data. A key limitation of this study is that the identified DDGIs are only potential interactions; As the dataset lacked clinical or genetic data, we could not determine whether actual adverse effects occurred. The clinical impact is likely lower than estimated, as individual phenotype determines whether a DGI is actionable. Misclassification of inhibitor and inducer status cannot be fully excluded due to reliance on external classification sources, although a conservative and transparent approach was applied to minimize this risk. Some DGIs may be attenuated by co-administered inducers or inhibitors. The choice of temporal windows also affects DDGI detection. For instance, antibiotics in small packages (eg ciprofloxacin, clarithromycin) likely contributed to short-window DDGIs involving OATP1B1 and CYP3A4/5. In contrast, long-term used drugs such as clopidogrel may explain more CYP2B6-related interactions in the 30-day window. Thus, in our case, we likely underestimated rather than overestimated the phenoconversion risk. By using both timeframes, we attempted to improve robustness by accounting for variability in dosing and package sizes. The use of claims data to establish drug use implies uncertainty regarding whether the medications claimed were taken, as these data do not strictly correspond to actual administration. Moreover, the Helsana database does not include information on OTC-drugs sold without a prescription. In this study, we considered ATC-codes also for drug combinations containing the interacting agent of interest. Fixed-dose combinations may complicate the interpretation of interaction risk, as co-formulated agents can influence each other or act on the same metabolic enzyme or transporter. In such cases, a combination product may include substances with opposing effects, for example an inhibitor and an inducer of the same pathway, leading to a net effect that differs from that expected for each drug administered alone. A comparable situation may also occur when the drugs are administered separately as the same interaction mechanisms apply regardless of formulation. When multiple interactions affect the same pathway, the resulting effect may deviate from the expected outcome of each interaction alone, making the overall clinical impact more difficult to predict.

Given the limited number of studies on the enzymes CYP3A4/5 and CYP2B6 or the transporters OATP1B1 and BCRP in this context, further studies are needed. A strength of our study is that Helsana includes a large and diverse range of individuals, making it reasonably representative of the general Swiss population. In addition, we used multiple sources to determine PGx drugs, inhibitors, and inducers. Interactions were stratified according to the application route (systemic or local) and the strength of the inhibitor or inducer to assess the clinical relevance of the interactions. In the future, it could be used to evaluate the priority of certain interactions for integration into a PGx clinical decision support system, with the goal of improving the prediction of a patient’s drug response phenotype. However, given the retrospective pharmacoepidemiological design and the absence of clinical or genetic outcome data, the actual clinical consequences and actionability of the identified DDGIs cannot be determined. Future research should therefore focus on validating the clinical relevance of the most prevalent DDGIs in real-world settings and assessing whether their targeted incorporation into clinical workflows enhances medication safety and therapeutic precision.^15^

## Conclusions

This study aimed to quantify the phenoconversion risk in the Swiss population for the enzymes CYP2B6 and CYP3A4/5 and the transporters OATP1B1 and BCRP. The present analysis highlights the advantages of integrating pharmacogenetics into clinical practice as a step toward personalized medicine. As new evidence is required to establish PGx preemptive testing as a standard procedure for new pharmacological treatments, this study contributes to a better understanding of currently underexplored DDGIs and supports the need for additional investigation into their clinical relevance. Other underestimated DDGIs involving less commonly studied enzymes or transporters should not be overlooked. These interactions warrant focused investigation, possibly incorporating specific PharmGKB recommendations, as they strongly influence the clinical relevance of DDGIs. Further research should prioritize assessing the real-world impact of such interactions, a process that requires access to genetic and clinical data.

## Data Availability

The datasets generated and/or analyzed during the current study are not publicly available due to confidentiality requirements issued by Helsana. Analysis codes and datasets can be made available by the corresponding author (s.allemann@unibas.ch) upon reasonable request and with permission of Helsana.

## Abbreviations

ABCG2, ATP Binding Cassette Subfamily G Member 2; ACR, American College of Rheumatology; ADR, Adverse Drug Reaction; AIOM, Italian Association of Medical Oncology; ATC, Anatomical Therapeutic Chemical; AusNZ, Australian and New Zealand consensus guidelines; BCRP, Breast Cancer Resistance Protein; CFF, Cystic Fibrosis Foundation; CPIC, Clinical Pharmacogenetics Implementation Consortium; CPNDS, Canadian Pharmacogenomics Network for Drug Safety; CYP, Cytochrome P450; DDI, Drug-Drug Interaction; DDGI, Drug-Drug-Gene Interaction; DGI, Drug-Gene Interaction; DPWG, Dutch Pharmacogenetics Working Group; FDA, U.S. Food and Drug Administration; N, Nervous System; N-drugs, Drugs Classified under the Nervous System Category (ATC Level 1); OATP1B1, Organic Anion Transporting Polypeptide 1B1; Obsan, Schweizerische Gesundheitsobservatorium; OTC, Over-The-Counter; PGx, Pharmacogenetics; PharmGKB, Pharmacogenomics Knowledge Base; RNPGx, French National Network of Pharmacogenetics; SEFF, Spanish Pharmacogenetics and Pharmacogenomics Society; SEOM, Spanish Society of Medical Oncology; SLCO1B1, Solute Carrier Organic Anion Transporter Family Member 1B1.

## References

[1] Cacabelos R, Naidoo V, Corzo L, Cacabelos N, Carril JC. Genophenotypic factors and pharmacogenomics in adverse drug reactions. *Int J Mol Sci*. 2021;22(24):13302. doi:10.3390/ijms222413302 34948113 PMC8704264

[2] Patton K, Borshoff DC. Adverse drug reactions. *Anaesthesia*. 2018;73(S1):76–12. doi:10.1111/anae.14143 29313907

[3] Dowd D, Williams G, VanDorn D, et al. Predicting drug-drug and drug-gene interactions in a community pharmacy population. *Am J Manag Care*. 2022;28(11):566–571. doi:10.37765/AJMC.2022.89259 36374614

[4] Oscarson M. Pharmacogenetics of drug metabolising enzymes: importance for personalised medicine. *Clin Chem Lab Med*. 2003;41(4):573–580. doi:10.1515/CCLM.2003.087 12747605

[5] Cascorbi I. Arzneimittelinteraktionen: prinzipien, Beispiele und klinische Folgen. *Dtsch Arztebl Int*. 2012;109(33–34):546–556. doi:10.3238/arztebl.2012.0546 23152742 PMC3444856

[6] Bank PCD, Swen JJ, Guchelaar HJ. Estimated nationwide impact of implementing a preemptive pharmacogenetic panel approach to guide drug prescribing in primary care in the Netherlands. *BMC Med*. 2019;17(1):1–14. doi:10.1186/s12916-019-1342-5 31196067 PMC6567386

[7] Klomp SD, Manson ML, Guchelaar HJ, Swen JJ. Phenoconversion of cytochrome P450 metabolism: a systematic review. *J Clin Med*. 2020;9(9):1–33. doi:10.3390/JCM9092890 PMC7565093 32906709

[8] Hahn M, Roll SC. The role of phenoconversion in the pharmacogenetics of psychiatric medication. *Pharmacogenomics*. 2023;24(9):485–487. doi:10.2217/PGS-2023-0100 37427432

[9] Wittwer NL, Meier CR, Huber CA, Schwabedissen HEMZ, Allemann S, Schneider C. Utilization of drugs with pharmacogenetic dosing recommendations in Switzerland: a descriptive study using the helsana database. *Pharmgenomics Pers Med*. 2022;15:967. doi:10.2147/PGPM.S382214 36447837 PMC9701506

[10] Lunenburg CATC, Hauser AS, Ishtiak-Ahmed K, Gasse C. Primary care prescription drug use and related actionable drug-gene interactions in the Danish population. *Clin Transl Sci*. 2020;13(4):798–806. doi:10.1111/cts.12768 32166845 PMC7359946

[11] Chanfreau-Coffinier C, Tuteja S, Hull LE, et al. Drug-drug-gene interaction risk among opioid users in the U.S. Department of Veterans Affairs. *Pain*. 2022;163(12):2390–2397. doi:10.1097/J.PAIN.0000000000002637 35319502

[12] Hahn M, Roll SC. The influence of pharmacogenetics on the clinical relevance of pharmacokinetic drug–drug interactions: drug–gene, drug–gene–gene and drug–drug–gene interactions. *Pharmaceuticals*. 2021;14(5):487. doi:10.3390/ph14050487 34065361 PMC8160673

[13] Mostafa S, Kirkpatrick CMJ, Byron K, Sheffield L. An analysis of allele, genotype and phenotype frequencies, actionable pharmacogenomic (PGx) variants and phenoconversion in 5408 Australian patients genotyped for CYP2D6, CYP2C19, CYP2C9 and VKORC1 genes. *J Neural Transm*. 2019;126(1):5–18. doi:10.1007/s00702-018-1922-0 30191366

[14] Gloor Y, Lloret-Linares C, Bosilkovska M, et al. Drug metabolic enzyme genotype-phenotype discrepancy: high phenoconversion rate in patients treated with antidepressants. *Biomed Pharmacother*. 2022;152:113202. doi:10.1016/j.biopha.2022.113202 35653884

[15] Blagec K, Kuch W, Samwald M. The importance of gene-drug-drug-interactions in pharmacogenomics decision support: an analysis based on austrian claims data. *Stud Health Technol Inform*. 2017;236:121–127. doi:10.3233/978-1-61499-759-7-121 28508787

[16] Whirl-Carrillo M, Huddart R, Gong L, Sangkuhl K, Thorn C, Whaley R. An Evidence-Based Framework for Evaluating Pharmacogenomics Knowledge for Personalized Medicine. *Clin Pharmacol Therap*; 2021;110(3):563–572. 34216021 10.1002/cpt.2350 PMC8457105

[17] Clinical Pharmacogenetics Implementation Consortium (CPIC). Available from: https://cpicpgx.org/. Accessed March 2, 2025.

[18] Dutch Pharmacogenetics Working Group (DPWG). Available from: https://www.knmp.nl/dossiers/farmacogenetica/pharmacogenetics. Accessed March 2, 2025.

[19] Guo C, Xie X, Li J, et al. Pharmacogenomics guidelines: current status and future development. *Clin Exp Pharmacol Physiol*. 2019;46(8):689–693. doi:10.1111/1440-1681.13097 31009088

[20] PharmGKB. Clinical guideline annotations. Available from: https://www.pharmgkb.org/guidelineAnnotations. Accessed April 30, 2026.

[21] Wittwer NL, Meier CR, Huber CA, et al. The prevalence of potential drug-drug-gene interactions: a descriptive study using swiss claims data. *Pharmgenomics Pers Med*. 2025;18:197–208. doi:10.2147/PGPM.S527556 40895400 PMC12392800

[22] Cleophas MC, Joosten LA, Stamp LK, Dalbeth N, Woodward OM, Merriman TR. ABCG2 polymorphisms in gout: insights into disease susceptibility and treatment approaches. *Pharmgenomics Pers Med*. 2017;10:129–142. doi:10.2147/PGPM.S105854 28461764 PMC5404803

[23] Daly AK. Pharmacogenetics and human genetic polymorphisms. *Biochem J*. 2010;429(3):435–449. doi:10.1042/BJ20100522 20626352

[24] Vermehren C, Nielsen RS, Jørgensen S, Drastrup AM, Westergaard N. Drug use among nursing home residents in Denmark for drugs having pharmacogenomics based (PGX) dosing guidelines: potential for preemptive PGX testing. *J Pers Med*. 2020;10(3):1–11. doi:10.3390/jpm10030078 PMC7565179 32752034

[25] Giorgetti A, Amurri S, Fazio G, et al. The evaluation of CYP2D6, CYP2C9, CYP2C19, and CYP2B6 phenoconversion in post-mortem casework: the challenge of forensic toxicogenetics. *Metabolites*. 2023;13(5):661. doi:10.3390/METABO13050661 37233702 PMC10221100

[26] Argevani L, Schuh MJ, Crosby S. Tacrolimus-induced bradykinesia secondary to phenoconversion in an elderly post-bilateral lung transplant patient. *Sr Care Pharm*. 2021;36(1):34–41. doi:10.4140/TCP.N.2021.34 33384032

[27] Bundesamt für Gesundheit (BAG). Die obligatorische Krankenversicherung (Ratgeber). Available from: https://www.bag.admin.ch/bag/de/home/versicherungen/krankenversicherung/krankenversicherung-das-wichtigste-in-kuerze.html. Accessed April 30, 2026.

[28] Schneider C, Schur N, Reinau D, Gut S, Schwenkglenks M. Helsana-drug-report; 2018. Available from: https://www.helsana.ch/en/helsana-group/media-publications/helsana-reports/drug-report.html. Accessed April 30, 2026.

[29] Sibylle T, Schur N, Wittwer NL, et al. Helsana report | drug report 2022 - helsana report. Available from: https://www.helsana.ch/en/helsana-group/media-publications/helsana-reports/drug-report.html. Accessed April 30, 2026.

[30] Whirl-Carrillo M, McDonagh EM, Hebert JM, et al. Pharmacogenomics knowledge for personalized medicine. *Clin Pharmacol Ther*. 2012;92(4):414–417. doi:10.1038/clpt.2012.96 22992668 PMC3660037

[31] PharmGKB. Clinical guideline annotations. Available from: https://www.pharmgkb.org/guidelineAnnotation/PA166265421. Accessed May 6, 2025.

[32] Zubiaur P, Fernández-Campos P, Navares-Gómez M, et al. Variants in comt, cyp3a5, cyp2b6, and abcg2 alterquetiapine pharmacokinetics. *Pharmaceutics*. 2021;13(10):1573. doi:10.3390/pharmaceutics13101573 34683865 PMC8540141

[33] Bakken GV, Rudberg I, Christensen H, Molden E, Refsum H, Hermann M. Metabolism of quetiapine by CYP3A4 and CYP3A5 in presence or absence of cytochrome B5. *Drug Metab Dispos*. 2009;37(2):254–258. doi:10.1124/DMD.108.023291 19022943

[34] Kim KA, Joo HJ, Lee HM, Park JY. Influence of ABCB1 and CYP3A5 genetic polymorphisms on the pharmacokinetics of quetiapine in healthy volunteers. *Pharmacogenet Genomics*. 2014;24(1):35–42. doi:10.1097/FPC.0000000000000020 24240480

[35] U.S. Food and Drug Administration. CYP enzyme- and transporter system-based clinical substrates, inhibitors, or inducers. Available from: https://www.fda.gov/drugs/drug-interactions-labeling/healthcare-professionals-fdas-examples-drugs-interact-cyp-enzymes-and-transporter-systems. Accessed January 30, 2025.

[36] Hôpitaux Universitaires de Genève. Interactions Medicamenteuses, Cytochromes P450 et p-Glycoproteine (pgp). Available from: https://www.hug.ch/sites/interhug/files/structures/pharmacologie_et_toxicologie_cliniques/images/carte_des_cytochromes_2020.pdf. Accessed February 7, 2025.

[37] Flockhart DA, Thacker D, McDonald C, Desta Z. The flockhart cytochrome P450 drug-drug interaction table. Available from: https://drug-interactions.medicine.iu.edu. Accessed February 8, 2025.

[38] Knox C, Wilson M, Klinger CM, et al. DrugBank 6.0: the DrugBank Knowledgebase for 2024. *Nucleic Acids Res*. 2024;52(D1):D1265–D1275. doi:10.1093/NAR/GKAD976 37953279 PMC10767804

[39] Park KW, Kang J, Park JJ, et al. Amlodipine, clopidogrel and CYP3A5 genetic variability: effects on platelet reactivity and clinical outcomes after percutaneous coronary intervention. *Heart*. 2012;98(18):1366–1372. doi:10.1136/heartjnl-2012-301892 22735685

[40] Zhou SF, Xue CC, Yu XQ, Li C, Wang G. Clinically important drug interactions potentially involving mechanism-based inhibition of cytochrome P450 3A4 and the role of therapeutic drug monitoring. *Ther Drug Monit*. 2007;29(6):687–710. doi:10.1097/FTD.0b013e31815c16f5 18043468

[41] Krasulova K, Holas O, Anzenbacher P. Influence of amlodipine enantiomers on human microsomal cytochromes p450: stereoselective time-dependent inhibition of CYP3A enzyme activity. *Molecules*. 2017;22(11):1–14. doi:10.3390/molecules22111879 PMC6150391 29099769

[42] Fedlex. Federal Act on Data Protection (FADP); 2019. Available from: https://www.fedlex.admin.ch/eli/cc/1993/1945_1945_1945/en. Accessed April 1, 2025.

[43] Schuler D, Roth S, Peter C. Psychopharmaka in der Schweiz: mengen, Kosten, wer sie bezieht und wer sie verschreibt; 2022. Available from: https://www.obsan.admin.ch/de/publikationen/2022-psychopharmaka-der-schweiz. Accessed April 1, 2025.

[44] Roe CM, McNamara AM, Motheral BR. Gender- and age-related prescription drug use patterns. *Ann Pharmacother*. 2002;36(1):30–39. doi:10.1345/APH.1A113 11816254

[45] Pazan F, Wehling M. Polypharmacy in older adults: a narrative review of definitions, epidemiology and consequences. *Eur Geriatr Med*. 2021;12(3):443–452. doi:10.1007/s41999-021-00479-3 33694123 PMC8149355

[46] Orlando V, Mucherino S, Guarino I, Guerriero F, Trama U, Menditto E. Gender differences in medication use: a drug utilization study based on real world data. *Int J Environ Res Public Health*. 2020;17(11):3926. doi:10.3390/ijerph17113926 32492925 PMC7312791

